# Leucine-Rich Repeat Kinase 2 Modulates Retinoic Acid-Induced Neuronal Differentiation of Murine Embryonic Stem Cells

**DOI:** 10.1371/journal.pone.0020820

**Published:** 2011-06-09

**Authors:** Cathrin Schulz, Marie Paus, Katharina Frey, Ramona Schmid, Zacharias Kohl, Detlev Mennerich, Jürgen Winkler, Frank Gillardon

**Affiliations:** 1 Boehringer Ingelheim Pharma GmbH & Co KG, CNS Research, Biberach an der Riss, Germany; 2 Division of Molecular Neurology, University Hospital Erlangen, Erlangen, Germany; National Institute of Health, United States of America

## Abstract

**Background:**

Dominant mutations in the *leucine-rich repeat kinase 2 (LRRK2)* gene are the most prevalent cause of Parkinson's disease, however, little is known about the biological function of LRRK2 protein. LRRK2 is expressed in neural precursor cells suggesting a role in neurodevelopment.

**Methodology/Principal Findings:**

In the present study, differential gene expression profiling revealed a faster silencing of pluripotency-associated genes, like *Nanog*, *Oct4*, and *Lin28*, during retinoic acid-induced neuronal differentiation of LRRK2-deficient mouse embryonic stem cells compared to wildtype cultures. By contrast, expression of neurotransmitter receptors and neurotransmitter release was increased in LRRK2+/− cultures indicating that LRRK2 promotes neuronal differentiation. Consistently, the number of neural progenitor cells was higher in the hippocampal dentate gyrus of adult LRRK2-deficient mice. Alterations in phosphorylation of the putative LRRK2 substrates, translation initiation factor 4E binding protein 1 and moesin, do not appear to be involved in altered differentiation, rather there is indirect evidence that a regulatory signaling network comprising retinoic acid receptors, let-7 miRNA and downstream target genes/mRNAs may be affected in LRRK2-deficient stem cells in culture.

**Conclusion/Significance:**

Parkinson's disease-linked LRRK2 mutations that associated with enhanced kinase activity may affect retinoic acid receptor signaling during neurodevelopment and/or neuronal maintenance as has been shown in other mouse models of chronic neurodegenerative diseases.

## Introduction

Parkinson's disease (PD) is the most prevalent movement disorder and is neuropathologically characterized by the selective loss of dopaminergic neurons in the substantia nigra related to motor dysfunction. Although the etiology of PD is incompletely understood, genetic studies have identified mutations in several genes that segregate with rare familial forms of the disease [1]. Mutations in the PARK8 gene encoding leucine-rich repeat kinase 2 (LRRK2) are the most prevalent cause of autosomal dominantly inherited PD and are characterised by typical brainstem Lewy body pathology. The most frequent mutation, LRRK2(G2019S), is found in the DF/YG motif of the kinase domain and is responsible for approximately 1% of sporadic PD and 5% of familial cases in Caucasians. LRRK2 is a 286 kDa protein containing an N-terminal leucine-rich repeat, a Ras of complex protein (Roc) GTPase domain, a C-terminal of Roc (Cor) region, a kinase domain, and a WD40 protein interaction domain. Several studies demonstrated that the G2019S mutation enhances kinase activity *in vitro* and that kinase activity mediates degeneration in transfected neurons [2], [3].

LRRK2 protein shows widespread, neuronal-specific expression in the adult mammalian brain [4]. Unexpectedly, LRRK2 knock-out mice do not exhibit any alterations in neuronal function or survival [5]. Therefore, the physiological function of LRRK2 in neurons remains enigmatic. Prominent LRRK2 expression has been detected in the subventricular zone and in the hippocampal dentate gyrus of the mouse brain suggesting a role of LRRK2 in neurogenesis [6], [7]. In the present study, wildtype and LRRK2-deficient mouse embryonic stem (ES) cells were used to investigate the effects of LRRK2 on neuronal differentiation.

## Methods

### Materials

Dulbecco's Modified Eagle Medium (DMEM), RPMI 1640, ES cell medium and fibroblast medium ingredients were obtained from Gibco (Invitrogen, Karlsruhe, Germany). Leukaemia inhibitory factor was purchased from Chemicon/Millipore (Schwalbach, Germany). Poly-D-lysine, gelatine, stocks for N2 and Complete medium, retinoic acid, mitomycin C, and EDTA were all obtained from Sigma (Steinheim, Germany). Laminin was supplied by Roche (Mannheim, Germany). Penicillin-streptomycin was purchased from Cambrex (North Brunswick, NJ, USA). Unless noted otherwise, all primary antibodies and all horseradish peroxidase-linked secondary antibodies were purchased from Cell Signaling Technology (Beverly, MA, USA). The rabbit anti-GluR5 antibody was supplied by Upstate Biotechnology (Lake Placid, NY, USA) and the mouse anti-GAPDH antibody was obtained from Biotrend Chemicals (Cologne, Germany). The rat anti-LRRK2 1E11 antibody was kindly provided by Prof. Marius Ueffing, Helmholtz Center (Munich, Germany). The monoclonal anti-stage-specific embryonic antigen-1 antibody was purchased from R&D Systems (Wiesbaden, Germany). Alexa Fluor 568-conjugated streptavidin was obtained from Invitrogen (Karlsruhe, Germany).

### Generation and differentiation of LRRK2+/− mouse embryonic stem cells

*LRRK2* knock-out mice were custom-generated by Artemis (Cologne, Germany) and will be described in detail elsewhere. Briefly, C57/BL6N mouse embryonic stem (ES) cells were transfected by electroporation, and homologous recombination of the targeting vector at the exon 2 region of the mouse genomic *LRRK2* locus was verified by Southern blot analysis (Figure S1). *LRRK2* mRNA expression was analysed by quantitative RT-PCR as described below. Since we wanted to exclude effects potentially arising from the neomycin resistance cassette in the present cell culture study, the marker was deleted after the selection of positive ES cell clones.

ES cells were cultured and differentiated according to the protocol previously described by others [8], [9]. Briefly, ES cells were cultured on mitomycin-inactivated mouse primary embryonic fibroblast feeder cells (StemCell Technologies, Grenoble, France) in ES cell medium (DMEM containing 15% (v/v) fetal bovine serum, 2 mM L-glutamine, 1 mM sodium pyruvate, 0.1 mM non-essential amino acids, 0.1 mM 2-mercaptoethanol, and 2000 U/ml leukaemia inhibitory factor) for at least two passages. Subsequently, ES cells were deprived of feeder by three passages on 0.2% gelatin-coated culture dishes in ES cell medium which was exchanged daily. Embryoid bodies (EBs) were formed from feeder-free ES cells grown as suspension culture on bacterial dishes (Greiner Bio-One, Solingen, Germany) in EB medium (ES cell medium lacking leukaemia inhibitory factor and containing 10% fetal bovine serum) for 8 days. Medium was exchanged on day 2, 4 and 6 of EB culture. Neuronal differentiation was induced by addition of 5 µM retinoic acid (RA) at day 4 and day 6 of EB culture. At day 8, EBs were dissociated in 0.05% (w/v) trypsin dissolved in phosphate-buffered saline (PBS) (AccuGene, Rockland, MA, USA) containing 0.04% (w/v) EDTA. Neural precursor cells obtained from dissociated EBs were plated in a density of 2×10^5^ cells per cm^2^ on dishes coated with poly-ornithin and laminin in N2 medium containing DMEM and Ham's nutrient mixture F12 1∶1 (v/v), L-glutamine (2 mM), insulin (25 µg mL^−1^), transferrin (50 µg mL^−1^), progesterone (20 nM), putrescine (100 nM), sodium selenite (30 nM) and bovine serum albumin (BSA, 50 µg mL^−1^). Medium was exchanged after 2 and 24 hours of plating. Forty-eight hours after plating of neural precursors, medium was changed to Complete medium composed of DMEM supplemented with alanin (2 µg mL^−1^), biotin (0.1 µg mL^−1^), carnitin (2 µg mL^−1^), ethanolamine (1 µg mL^−1^), galactose (15 µg mL^−1^), proline (7.8 µg mL^−1^), putrescine (16 µg mL^−1^), sodium pyruvate (25 µg mL^−1^), sodium selenite (0.02 µg mL^−1^), vitamin B12 (0.3 µg mL^−1^), zinc sulphate (0.2 µg mL^−1^), catalase (2.6 µg mL^−1^), glutathione (1 µg mL^−1^), superoxide dismutase (2.5 µg mL^−1^), linoleic acid (1 µg mL^−1^), linolenic acid (1 µg mL^−1^), progesterone (6.3 ng mL^−1^), retinol (0.1 µg mL^−1^), retinyl acetate (0.1 µg mL^−1^), tocopherole (1 µg mL^−1^), tocopherole acetate (1 µg mL^−1^), BSA (2.5 mg mL^−1^), transferrin (5 µg mL^−1^), insulin (4 µg mL^−1^) and L-glutamine (2 mM). The medium was exchanged at day 4 of differentiation. At all stages of ES cell differentiation the incubator settings were kept at 36.5°C, 7% CO_2_ and 90% humidity.

### Protein extraction and Western blot analysis

At distinct stages of differentiation, cells were harvested in lysis buffer including 30 mM Tris-HCl (pH 8.5), 4% (v/v) Chaps, 8 M urea, and phosphatase inhibitor cocktail (Sigma, Germany). Soluble protein supernatant fractions were obtained by centrifugation 12,500× g for 10 minutes at 10°C. Proteins were separated by SDS-PAGE and transferred to nitrocellulose membranes (Protran, Whatman, Dassel, Germany). Membranes were routinely checked for protein load and protein transfer using a protein staining kit (MemCode, Pierce, Rockford, Illinois, USA) that reversibly stains for total protein. Blots were blocked for 1 h in Tris-buffered saline containing 0.1% (v/v) Tween-20 and 5% (w/v) non-fat dry milk. Membranes were subsequently incubated over night at 4°C with primary antibodies (1 µg mL^−1^). After repeated washings with Tris-buffered saline/Tween-20 (0.1% v/v), membranes were incubated for 1 h at room temperature with the corresponding secondary antibody (0.5 µg mL^−1^). Antibody binding was detected by enhanced chemiluminescence staining (ECL Western blotting analysis system, GE Healthcare, Munich, Germany) and exposure to X-ray films. Scanned Western blot bands were quantified densitometrically and values were normalized to the amount of glyceraldehyde 3-phosphate dehydrogenase (GAPDH) using Quantity One software (Imaging Densitometer, Model GS-700, Biorad, Munich, Germany).

### RNA isolation

For total RNA isolation, ES cells splitted for 3 passages on gelatine and ES cell-derived neurons differentiated for 7 days (triplicate cultures of each genotype) were lysed in RLT buffer provided by a ready-to-use RNA isolation kit (RNAeasy Kit, Qiagen, Hilden, Germany). Sample homogenisation, total RNA isolation and on-column DNase digest were performed according to the manufacturer's instruction. RNA concentration/purity was determined on a spectrophotometer (NanoDrop ND-1000), and RNA quantity/quality was analysed on a microfabricated chip (RNA 6000 Nano LabChip kit, Agilent Technologies, Böblingen, Germany). The RNA chip assay was executed on a microfluidics-based platform (Agilent 2100 bioanalyzer) in accordance to the manufacturer's recommendation.

### Microarray gene expression profiling

Gene expression profiling was performed using the GeneChip Mouse Genome 430 2.0 Array (Affymetrix, Santa Clara, CA, USA) according to the manufacturer's instruction. One-cycle synthesis of double-stranded cDNA was done using 2 µg of isolated total RNA. After amplification and biotin-labelling of cDNA followed by *in vitro* transcription, biotin-labeled cRNA was purified, fragmented by heating and hybridized to the arrays (triplicates for each genotype). Arrays were scanned using a Hewlett-Packard GeneArray Scanner and data were analysed by Affymetrix GeneChip software [10]. Data have been processed with the R Language and Environment for Statistical Computing (R) 2.5.0 (www.r-project.org) in combination with Bioconductor 2.0 [11]. The Bioconductor affy package [12] has been used for quality control and data were normalized using the robust multi-array average (rma) [13] expression measure. Linear models [14] as implemented in the Bioconductor limma package [15] were applied to calculate fold changes, the resulting p-values were FDR-corrected [16]. Genes showing ≥1.5-fold changes in expression levels and an adjusted p-value<0.01 as a specific hybridization signal were considered deregulated. Data are publicly available from ArrayExpress database [17] (www.ebi.ac.uk/aerep/login), experiment E-MEXP-2963. To analyze the enrichment of deregulated gene transcripts in canonical pathways Ingenuity Pathway Analysis 8.8. software (Ingenuity Systems, Redwood City, Califormia, USA) was utilized. All genes that are differentially regulated with a p-value<0.01 and an absolute fold change >1.5 when comparing LRRK2-deficient to wildtype cells were considered for analysis. The significance of the association between the gene transcript and the canonical pathway was measured in two ways: First, a ratio of the number of molecules from the data set that map to the pathway divided by the total number of molecules that are annotated to the pathway was calculated. Second, Fisher's exact test was conducted to calculate a p-value determining the probability that the association between the genes in the dataset and the canonical pathway is explained by chance alone.

### Quantitative RT-PCR

Lab equipment and chemicals used for LRRK2 mRNA expression level analyses were purchased from Applied Biosystems (Darmstadt, Germany). Primer Express Software Version 3.0 assisted in the design of primers and probe. Expression levels of mRNA were quantified via one-step RT-PCR (TaqMan) in a 10 µL reaction containing QuantiTect Probe RT-PCR Master Mix (Qiagen, Hilden, Germany), forward and reverse primer (0.4 µM each), 5′ fluorescence-labelled probe (0.2 µM), QuantiTect Multiplex RT Mix (0.5 µL per reaction, Qiagen, Hilden, Germany) and RNA template (100 ng). The following primer and probes were applied: mouse LRRK2-forward (ACATGCTGGTGTTCACCTACTC), mouse LRRK2-reverse (CAACAGAGGCACGTGGAAATTTT), mouse LRRK2-probe (5′ FAM-labelled MGB probe, ACCGCGCCTCCAAGTT). The reaction was carried out in optical 96 well-plates. The thermocycler (7500 Fast Real-Time PCR System, 7500 Fast System Software v1.3.1) was programmed for reverse transcription (30 min at 48°C), a single denaturation step (10 min at 95°C) and 40 amplification cycles (15 s at 95°C cDNA denaturation, 60 s at 60°C combined annealing/extension and fluorescence data collection step). Real-Time PCR was carried out in triplicate for each marker and sample.

### Immunocytochemistry

Cells grown either on Lab-Tek chamber slides (Nunc, Wiesbaden, Germany) or 96-well plates (Becton Dickinson, Heidelberg, Germany) were fixed in ice-cold paraformaldehyde (4%) for 10 min, washed in PBS and blocked for 1 h in PBS containing 1% BSA and 0.1% saponin (Sigma, Steinheim, Germany). Cells were then incubated with primary antibodies (1 µg mL^−1^) in blocking solution over night at 4°C. After washing with PBS, cells were incubated with the secondary antibodies (5 µg mL^−1^) for 1 h at 37°C. When working with the biotin-streptavidin system, cells were again rinsed with PBS and then incubated for 1 h at 37°C with Alexa Fluor-568 conjugated streptavidin (Invitrogen, Karlsruhe, Germany). After washing with PBS, cells were mounted in Confocal Matrix (Micro-Tech-Lab, Graz, Austria). Stainings for alkaline phosphatase were performed using a commercially-available detection kit (Chemicon/Millipore, Schwalbach, Germany) according to the manufacturer's protocol. Images were collected on an inverted microscope (Axiovert 135, Zeiss, Goettingen, Germany). Alternatively, cells were imaged using a Becton Dickinson Pathway 435 imager and evaluated using AttoVision 1.5 software (Becton Dickinson Biosciences, Rockville, Maryland, USA).

### Kinase assay in solution

N-terminal GST-tagged human LRRK2 (amino acids 970-2527) (Invitrogen, Carlsbad, California, USA) was incubated in kinase reaction buffer (25 mM Tris, pH 7.5, 10 mM MgCl_2_, 0.1 mM EGTA, 2 mM dithiothreitol, 1 mM sodium orthovanadate) containing 100 µM ATP and 30 µCi [γ-^33^P]ATP at 30°C for 45 minutes. The enzyme reaction was stopped by the addition of 4× Laemmli buffer (4% sodium dodecyl sulfate, 400 mM dithiothreitol, 200 mM Tris, pH 7.5). Proteins were heated for 10 minutes to 70°C, and separated on 4–12% NuPAGE Bis-Tris gradient minigels. Gels were dried for 30 minutes at 70°C in a vacuum gel dryer (Model 583, Bio-Rad, Munich, Germany) and exposed to phosphoscreens over night. Phosphoscreens were imaged on a Typhoon 9400 laser scanner (GE Healthcare, Freiburg, Germany) and quantitated using Quantity One (Bio-Rad, Munich, Germany). Phosphosite mapping of recombinant retinoic acid receptor alpha was performed by NextGen Sciences (Ann Arbor, Michigan, USA) as has been described elsewhere [18]. Recombinant full-length retinoic acid receptor alpha and retinoic acid receptor beta was purchased from Abnova (Taipei, Taiwan) and tested at the concentrations indicated in the Results section.

### Neurotransmitter release

For measurement of depolarisation-induced neurotransmitter release, ES cell-derived neurons differentiated for 7 days (triplicate cultures of each genotype) were cultured in medium containing 80 mM KCl for 3 min. The medium was harvested in ice-cold 0.1 M perchloric acid and stored at −80°C. GABA and glutamate content were analysed by HPLC and electrochemical detection at Brains On-Line (Groningen, Netherlands) using published protocols (www.brainsonline.org).

### Hippocampal neurogenesis in LRRK2-deficient mice

All animal experiments were carried out in accordance with the National Institutes of Health guidelines for the treatment of laboratory animals and the European Communities Council Directive (86/609/EEC) and were approved by the local governmental commission for animal health. To label proliferating hippocampal stem cells, BrdU (100 mg/kg body weight) was administered to LRRK2 knockout mice and wildtype littermate controls (n = 6 per group) by a single intraperitoneal injection. After 24 hours animals were deeply anaesthetized and transcardially perfused with 0.9% NaCl followed by 4% paraformaldehyde in 0.1 M phosphate-buffered saline (PBS) (pH 7.4). Brains were removed, postfixed overnight in 4% paraformaldehyde at 4°C, and stored in 30% sucrose in 0.1 M PBS at 4°C. To label surviving/differentiating hippocampal progenitor cells, BrdU was administered to a second goup of mice (n = 6 per genotype) from day 1 to day 5 by daily intraperitoneal injections (50 mg/kg body weight). Anaesthetized animals were perfused on day 28 of treatment and brains were processed as described above.

Immunohistochemical analysis was performed as has been published elsewhere [19]. Brains were cut into 25 µm coronal sections. For BrdU immunostaining free-floating sections were pretreated with formamide and HCl to denature DNA. Thereafter, sections were incubated with 0.6% H_2_O_2_, blocked in 3% donkey serum, and permeabilised with 0.1% Triton-X100. The sections were incubated with either a rat monoclonal anti-BrdU antibody (1∶500, AbD Serotec, Oxford, UK) or a goat polyclonal anti-doublecortin (DCX) antibody (1∶500; Santa Cruz Biotechnology, Santa Cruz, California, USA). Brain sections were subsequently incubated with a biotinylated secondary antibody (1∶1000) followed by avidin-biotin-peroxidase complex (1∶100) and diaminobenzidine as substrate (Vector Laboratories, Burlingame, California, USA). Sections were mounted on Superfrost Plus slides (Menzel, Braunschweig, Germany) in Neo Mount medium (Merck, Darmstadt, Germany). All counting procedures were performed on coded slides. To count BrdU- and DCX-immunopositive cells, every 6th hippocampal section was analysed using a light microscope (AxioImager M2, Zeiss, Jena, Germany) equipped with a semi-automatic stereology system (Stereoinvestigator, MicroBrightField, Magdeburg, Germany). The total number of positive cells per dentate gyrus was calculated using the optical dissector method [20]. Control stainings where the primary antibody was omitted produced no signal.

### Statistical analysis

All experiments were performed using at least three biological replicates. Mean values ± standard deviations are indicated in the Results section. Statistical significance was determined by two-tailed Student's t-test. A p-value<0.05 was considered significant.

## Results

### Characterisation of LRRK2+/− murine embryonic stem cells during neuronal differentiation

**Figure 1:**
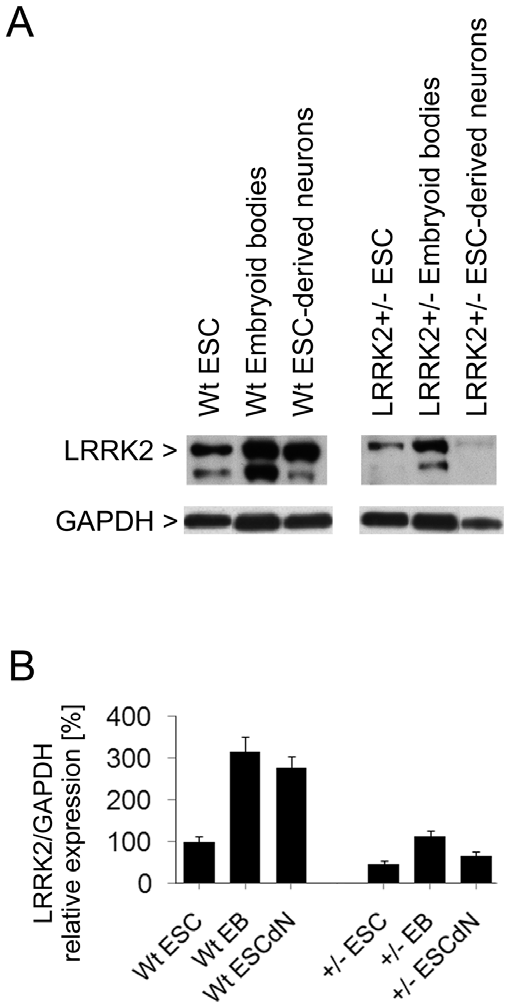
Western blot analysis of LRRK2 protein levels in cell lysates. (**A**) LRRK2 (and one fragment) is expressed in cultured mouse embronic stem cells (ESC), and its expression increases during retinoic acid-induced neuronal differentiation. LRRK2 levels in LRRK2+/− cells are about 50% lower than in wildtype (wt) cells. Levels of glyceraldehyde 3-phosphate dehydrogenase (GAPDH) were co-detected as gel loading controls and indicate that lower protein amounts were loaded in the LRRK2+/− ESC-derived neuron sample. The immunoblots shown are representative of three independent experiments. (**B**) Full-length LRRK2 protein bands were quantified by densitometry and normalized to GAPDH loading control using Quantity One software. Bars represent mean ± SD (n = 3).

By homologous recombination of the targeting vector at the exon 2 region of the mouse genomic LRRK2 locus, heterozygous LRRK2*+/−* C57BL/6N mouse embryonic stem (ES) cells were generated. Two ES cell clones, where homologous recombination was verified by Southern blot analysis (Figure S1), were used for further characterisation. Quantitative RT-PCR revealed a significant reduction of LRRK2 mRNA levels to 55.9±4.3% compared to wildtype C57BL/6N ES cells. We also analysed expression levels of LRRK2 protein at different stages of neuronal differentiation by Western blotting using a validated anti-LRRK2 antibody. Densitometric quantification of the 275 kDa full-length LRRK2 band indicated that LRRK2 protein is expressed in cultured murine ES cells (Figure 1). Protein levels significantly increased in embryoid bodies and remained elevated in ES cell-derived neurons. In line with the LRRK2 mRNA data mentioned above, LRRK2 protein levels in LRRK2+/− cells were about 50% lower than in wildtype cells at all stages (Figure 1). Transduction of LRRK2-deficient embryonic ES cells with an adenoviral type 5 expression vector in order to reconstitute LRRK2 expression caused cell death as has been shown in other cell culture models overexpressing LRRK2 (data not shown).

**Figure 2:**
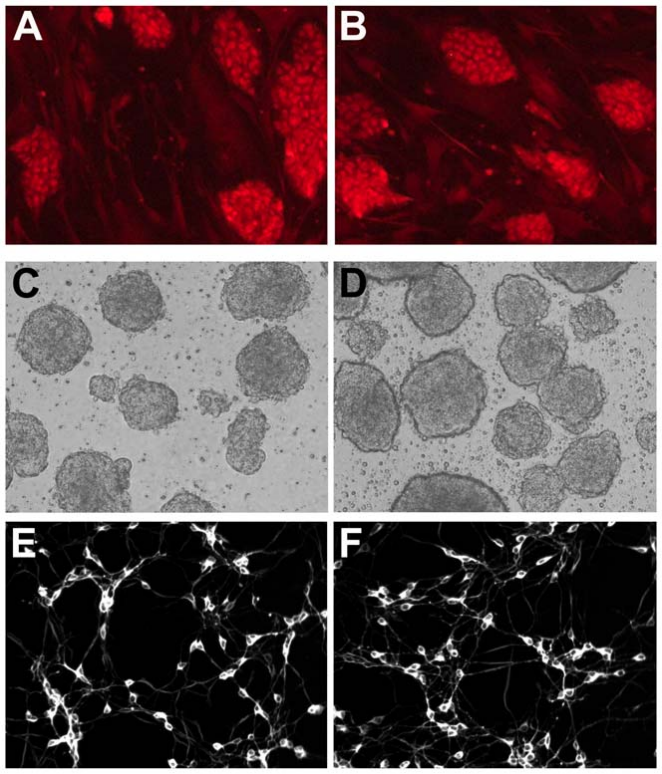
Immunocytochemical and morphological analysis of LRRK2+/− (right panels) and wildtype (left panels) ES cells during neuronal differentiation. (**A, B**) Undifferentiated ES cells on fibroblast feeders are immunoreactive for the pluripotency marker stage-specific embryonic antigen-1. (**C, D**) Embryoid bodies grown in suspension culture show similar morphology. (**E, F**) At differentiation day 7, ES cell-derived neurons immunostained for β-III tubulin exhibit similar cell densities and neurite networks.

Pluripotency of LRRK2+/− and wildtype ES cells was confirmed both by detection of high alkaline phosphatase activity [18] and by immunostaining for the surface marker stage-specific embryonic antigen-1 (SSEA-1) (Figure 2 A, B). During all steps of differentiation no obvious differences in cell density or morphology could be observed between *LRRK2+/−* and wildtype cells by microscopical imaging and digital analysis (Figure 2). Immunostaining for neuron-specific β-III tubulin and nuclear Hoechst 33342 labelling revealed that about 95% of both *LRRK2+/−* and wildtype cells adopted a neuronal phenotype after retinoic acid treatment for 7 days (Figure 2) [18]. Neuronally-differentiated ES cells cultured for >7 days exhibited neurite blebbing precluding longer observation times.

### Microarray gene expression analysis

**Figure 3:**
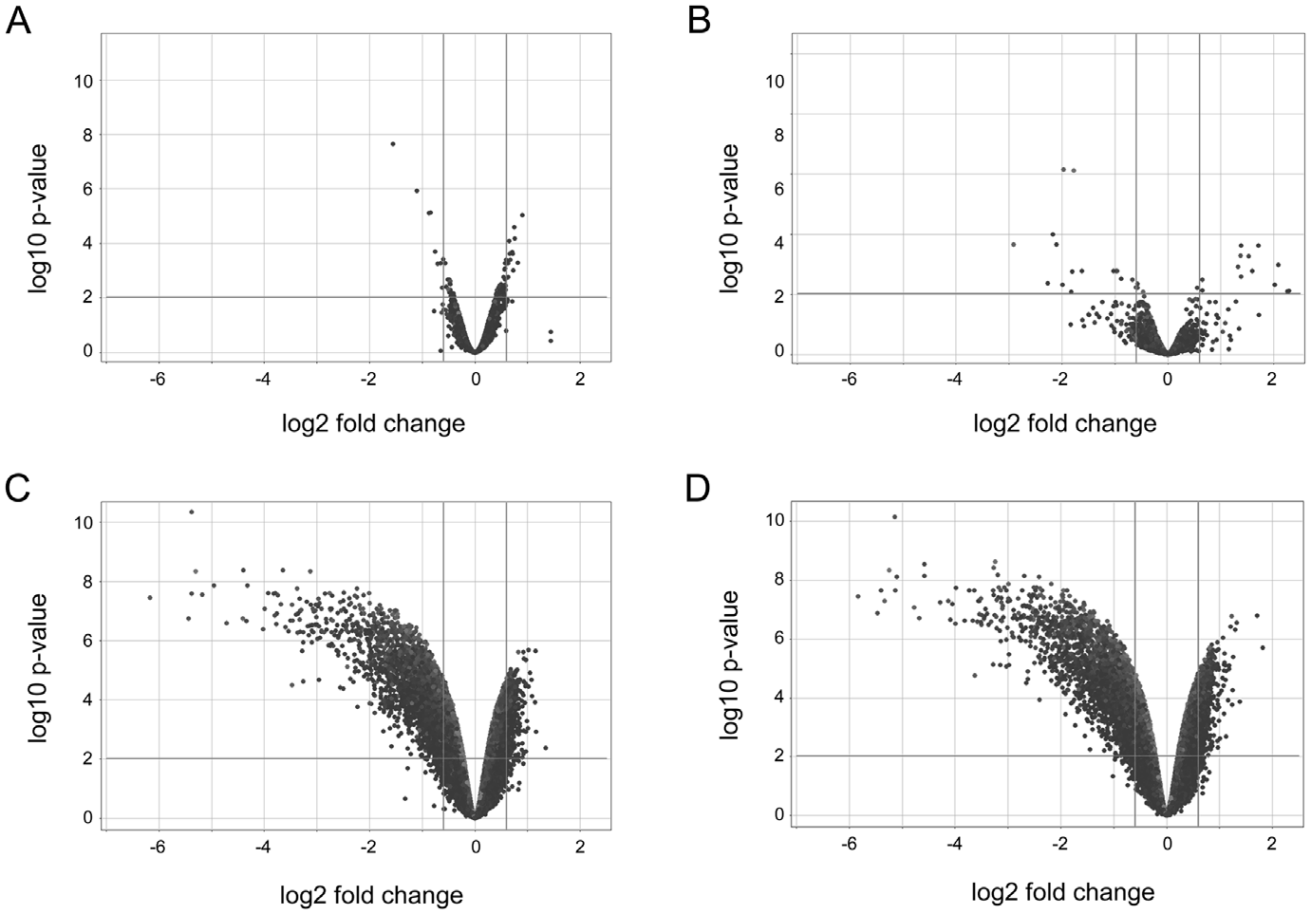
Affymetrix GeneChip analysis comparing mRNA expression levels in: (A) clone A LRRK2+/− ES cells versus wildtype ES cells, (C) clone A LRRK2+/− ES cell-derived neurons versus wildtype ES cell-derived neurons, (D) clone B LRRK2+/− ES cell-derived neurons versus wildtype ES cell-derived neurons, and (B) clone A LRRK2+/− ES cell-derived neurons versus clone B LRRK2+/− ES cell-derived neurons. Massive changes in gene expression become detectable in LRRK2+/− ES cell-derived neurons compared to wildtype ES cell-derived neurons 7 days following neuronal differentiation by retinoic acid administration.

To get more insight into the cellular function of LRRK2, we performed differential gene expression profiling using RNA samples from undifferentiated, SSEA1-immunopositive ES cells and from differentiated, β-III tubulin-immunoreactive ES cell-derived neurons, respectively. As shown in Figure 3, only 9 genes were significantly (fold change ≥1.5 and adjusted p-value≤0.01) down-regulated and 14 genes were up-regulated in LRRK2+/− ES cells compared to wildtype controls. By contrast, 7 days after starting retinoic acid-induced differentiation, 2404 genes were significantly down-regulated and 575 genes were up-regulated in LRRK2+/− ES cell-derived neurons compared to wildtype cultures. Similar changes were detected when two clones (A, B) with monoallelic LRRK2 deletion were compared to wildtype ES cell-derived neurons, whereas gene expression did not differ between the LRRK2+/− clones A and B (Figure 3). The complete Genechip dataset has been loaded into ArrayExpress database (www.ebi.ac.uk/aerep/login) under experiment E-MEXP-2963. These findings indicate that LRRK2 adopts a critical biological function during retinoic acid-induced neuronal differentiation, although LRRK2 is already expressed at the ES cell stage.

Notably, mRNA expression of the key pluripotency transcription factors Nanog, Lin28, and Oct4 (also termed Oct3 and Pou5f1) was significantly reduced in LRRK2+/− ES cells 7 d after retinoic acid-induced neuronal differentiation (Table 1). Moreover, expression of pluripotency-linked, downstream target genes of Nanog and Oct4 in ES cells, like developmental pluripotency-associated protein, left-right determination factor 2, secreted phosphoprotein, F-box protein-15, and zinc-finger protein-42 [21], [22], declined as well (Table 1). On the other hand, genes that are transcriptionally repressed by Nanog/Oct4 and that promote differentiation (e.g. homeobox B5, homeobox A4, Pou4f2) showed a moderate increase in expression in LRRK2+/− ES cell-derived neurons (Table 1) suggesting that differentiation may be accelerated in retinoic acid-treated LRRK2+/− ES cells. Consistently, mRNA levels of various ion channels and neurotransmitter receptors, like voltage-gated sodium channel Scn1a, voltage-gated potassium channel Kcnq2, metabotropic glutamate receptor 8, and ionotropic glutamate receptor 1, were significantly higher in LRRK2+/− neurons (Table 1). Messenger RNA expression of glial markers (e.g. glial fibrillary acidic protein) did not differ between LRRK2+/− and wildtype cell cultures indicating that there is no redirection in differentiation from neuronal to glial lineages. Ingenuity canonical pathway analysis also points to an enrichment of gene transcripts annotated to stem cell pluripotency, cell cycle regulation, and neuronal function (Figure S2).

**Table 1:** Differentially-expressed genes in LRRK2+/− versus wildtype embryonic stem cell-derived neurons.

SYMBOL	ACCESSION	GENE	fold change	adj. p-value
Nanog	NM_028016.2	Nanog homeobox	−44.359	1.3E-07
Pou5f1	NM_013633.2	POU domain, class 5, transcription factor 1	−34.983	2.2E-08
Spp1	NM_009263.1	secreted phosphoprotein 1	−23.884	2.8E-09
Lin28	NM_145833.1	lin-28	−23.555	7.1E-09
Dppa2	NM_028615.1	developmental pluripotency associated 2	−15.776	1.8E-08
Lefty2	NM_177099.3	Left-right determination factor 2	−14.112	2.4E-07
Eif4ebp1	NM_007918.3	eukaryotic translation initiation factor 4E binding protein 1	−8.790	2.2E-08
Rai14	NM_030690.2	retinoic acid induced 14	−5.756	4.8E-07
Crabp2	NM_007759.2	cellular retinoic acid binding protein II	−3.166	1.9E-06
Msn	NM_010833.2	moesin	−2.579	1.7E-06
Slc1a6	NM_009200.2	solute carrier family 1 (aspartate/glutamate transporter)	1.517	2.6E-03
Cend1	NM_021316.3	cell cycle exit and neuronal differentiation 1	1.530	1.8E-04
Snca	NM_009221.2	synuclein, alpha	1.598	1.3E-04
Slc6a11	NM_172890.3	solute carrier family 6 (neurotransmitter transporter, GABA)	1.612	6.3E-05
Kcnma1	NM_010610.2	potassium large conductance calcium-activated channel	1.614	7.8E-04
Gabbr1	NM_019439.3	gamma-aminobutyric acid (GABA-B) receptor, 1	1.631	2.8E-05
Syn2	NM_013681.1	synapsin II	1.634	3.6E-05
Cacna2d3	NM_009785.1	calcium channel, voltage-dependent, alpha2/delta subunit 3	1.821	3.0E-06
Grm8	NM_008174.2	glutamate receptor, metabotropic 8	1.845	6.8E-06
Grik1	NM_010348.2	glutamate receptor, ionotropic, kainate 1	2.073	2.1E-05
Nrn1	NM_153529.1	neuritin 1	2.134	6.3E-06
Sncg	NM_011430.2	synuclein, gamma	2.266	1.1E-05
Calb2	NM_007586.1	calbindin 2	2.396	4.0E-06

The actin-binding proteins ezrin/radixin/moesin and the translational repressor eukaryotic translation initiation factor 4E binding protein 1 (4E-BP1) have been identified as LRRK2 substrates *in vitro* [23], [24]. Our Genechip analysis revealed that mRNA expression of moesin and 4E-BP1 was significantly down-regulated in LRRK2+/− ES cell-derived neurons (Table 1).

### Western blot analysis

**Figure 4:**
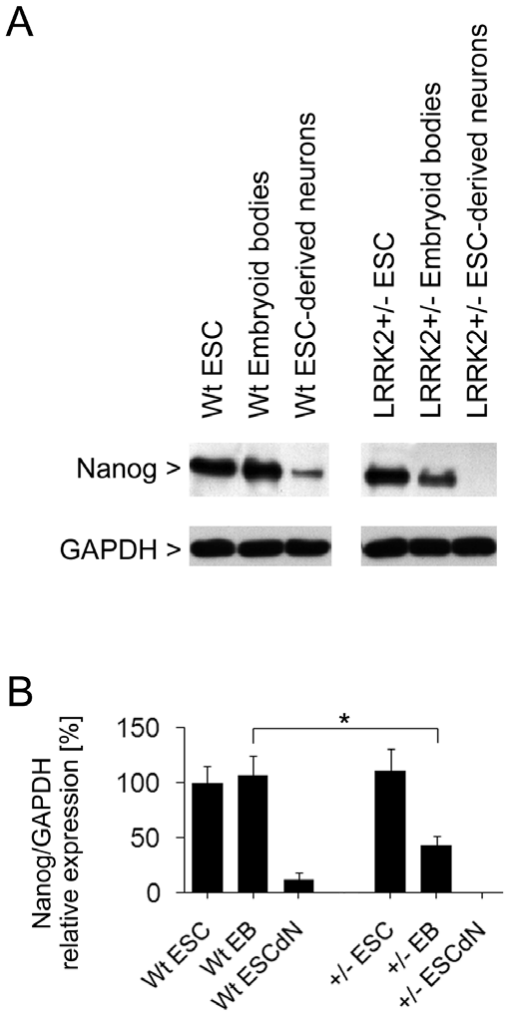
Western blot analysis of Nanog protein levels in cell lysates. (**A**) Protein expression of the pluripotency marker Nanog decreases more rapidly during retinoic acid-induced neuronal differentiation in LRRK2+/− cells. Levels of glyceraldehyde 3-phosphate dehydrogenase (GAPDH) were co-detected as gel loading controls. The immunoblots shown are representative of three independent experiments. (**B**) Nanog immunoreactive bands were quantified by densitometry and normalized to GAPDH loading control. Bars represent mean ± SD (n = 3). Asterisk (*) indicates statistical significant (p<0.05) difference.

**Figure 5:**
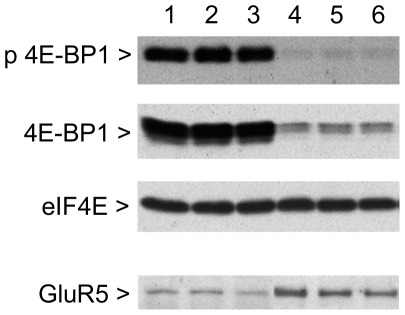
Protein expression and phosphorylation of the putative LRRK2 substrate 4E binding protein 1 (4E-BP1). Both signals decline in LRRK2+/− ES cell-derived neurons (lane 4–6) compared to wildtype cultures (lane 1–3). Protein levels of eukaryotic translation initiation factor 4E (eIF4E), the interaction partner of 4E-BP1, are unchanged. Protein expression of the ionotropic glutamate receptor 5 (GluR5) is increased in LRRK2+/− neurons (lane 4–6) compared to wildtype cells (lane 1–3). Three replicate cultures per genotype were analysed. For densitometric quantification see Results section.

**Figure 6:**
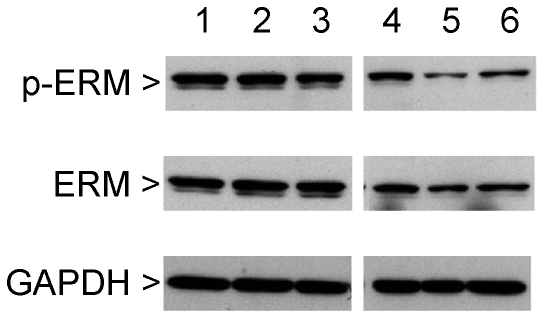
Protein expression and phosphorylation of the putative LRRK2 substrate proteins ezrin, radixin, and moesin (ERM). Immunoreactive bands are reduced in LRRK2+/− ES cell-derived neurons (lane 4–6) compared to wildtype cultures (lane 1–3). Levels of glyceraldehyde 3-phosphate dehydrogenase (GAPDH) are shown as gel loading controls. Three replicate cultures per genotype were analysed. For densitometric quantification see Results section.

In order to confirm alterations in mRNA expression on the protein level, protein extracts from cultured cells were assessed by Western blotting. The pluripotency transcription factor Nanog was still detectable in extracts from wildtype neurons 7 d after retinoic acid-induced differentiation, whereas Nanog protein was not detected in LRRK2+/− neurons (Figure 4). On the other hand, protein levels of the ionotropic glutamate receptor 5 were significantly increased by 117.5±9.1% (Figure 5). Additionally, Western blotting revealed a decline in ezrin/radixin/moesin protein levels by 52.3±6.1% (Figure 6) and in 4E-BP1 by 81.0±7.7% in LRRK2+/− ES cell-derived neurons, whereas protein expression of eukaryotic translation initiation factor 4E (eIF4E), the interaction partner of 4E-BP1, remained unaltered (Figure 5).

Since LRRK2 has been shown to phosphorylate ezrin/radixin/moesin on a conserved threonine residue (Thr588 in moesin) and 4E-BP1 on threonine 70 [23], [24], we used phospho-specific antibodies to investigate alterations in their phosphorylation status in LRRK2+/− neurons derived from ES cells. Unexpectedly, neither the ratio of phospho-ezrin/radixin/moesin(Thr588) to total ezrin/radixin/moesin nor the ratio of phospho-4E-BP1(Thr70) to total 4E-BP1 showed a significant decrease in LRRK2+/− neurons indicating that LRRK2 is not rate-limiting for phosphorylation in our cell culture model (Figures 5 and 6). Membrane staining for total protein revealed that similar amounts of protein were loaded per lane (Figure S3).

### Retinoic acid receptor signaling

**Figure 7:**
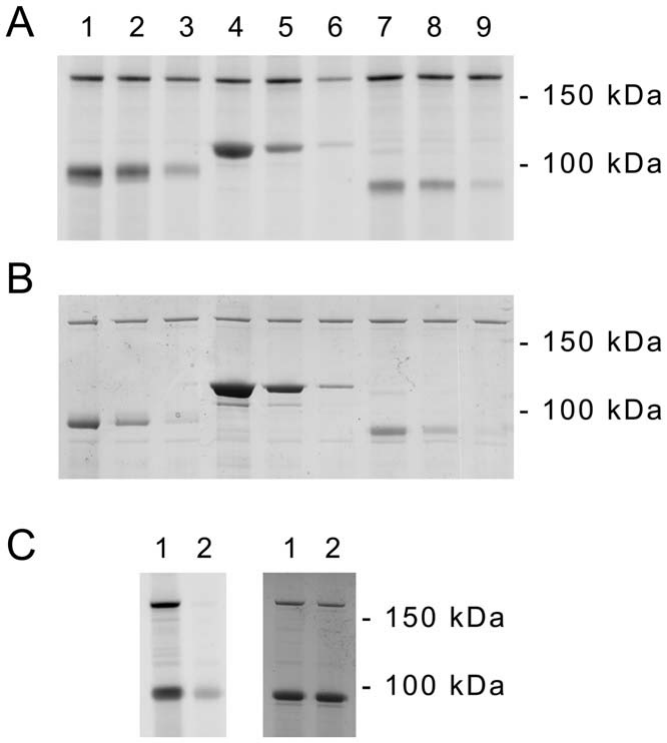
Retinoic acid receptor alpha is efficiently phosphorylated by recombinant LRRK2. (**A**) Autoradiogram demonstrating phosphorylation of recombinant tubulin-beta 2C (lane 1–3), heat-treated meosin (lane 4–6), and retinoic acid receptor alpha (lane 7–9) by recombinant LRRK2(G2019S) (amino acids 970-2527). Three-fold dilutions of each recombinant protein were incubated with kinase and [γ-^33^P]ATP for 45 min. (**B**) Coomassie Blue protein post-staining of the gel. (**C**) (Left panel) Kinase-dead LRRK2(D1994A) does not autophosphorylate or phosphorylate retinoic acid receptor alpha (lane 2) compared to active LRRK2(G2019S) (lane 1). (Right panel) Similar protein amounts are detected by Coomassie Blue protein staining.

Gene expression profiling also provided a first hint that retinoic acid signaling may be altered in LRRK2+/− neurons during differentiation from ES cells. As shown in Table 1, several retinoic acid receptor target genes (e.g. retinoic acid induced 14, stimulated by retinoic acid gene 8, cellular retinoic acid binding protein 2) were differentially expressed, while mRNA levels of retinoic acid receptors alpha and beta were unaltered. Transcriptional regulation by retinoic acid receptors is modulated by various kinases (e.g. MAP kinases) through receptor phosphorylation thereby modulating binding to coactivators, repressors, or DNA [25]. This led us to investigate whether LRRK2 is able to phosphorylate retinoic acid receptors *in vitro* and to compare efficiency of phosphorylation of retinoic acid receptor to moesin and tubulin-beta 2C which are potently phosphorylated by recombinant LRRK2 [18]. Using threefold dilutions of each recombinant protein substrate we observed that retinoic acid receptors alpha (Figure 7 A, B) and beta (not shown) become phosphorylated by LRRK2 as efficiently as moesin and tubulin-beta 2C, respectively. As a negative control, kinase-dead LRRK2(D1994A) did not phosphorylate either protein under the same assay conditions (Figure 7C). By tandem mass spectrometry one phosphorylated peptide (DGGGLAPPPGSCSPSLSPSSNR) was detected in a tryptic digest of retinoic acid receptor alpha (63% sequence coverage), and serine-445 was identified as phosphorylation site.

### Neurotransmitter levels

To demonstrate biological relevance of the increased expression of neuronal markers in LRRK2+/− ES cell-derived neurons, we assessed neurotransmitter release during potassium-induced depolarisation. Neurotransmitter concentrations in the cell culture medium were analysed by HPLC and normalised to protein amounts in ES cell lysates. GABA concentrations were significantly higher in LRRK2+/− neuronal cultures compared to wildtype controls (82.0±3.3 versus 58.3±2.8 nmol/g protein, LRRK2-deficient versus wildtype cultures, mean ± SD, n = 3 each), and glutamate concentrations showed a trend towards an increase (9.5±0.8 versus 8.5±0.1 µmol/g protein). These findings further substantiate an accelerated neuronal differentiation in retinoic acid-treated LRRK2+/− ES cells.

### Hippocampal neurogenesis in adult LRRK2-deficient mice

To analyse the impact of LRRK2 deficiency on neurogenesis in vivo, we investigated proliferation and differentiation of hippocampal stem cells in the subgranular zone of the dentate gyrus of adult mice. The number of proliferating BrdU-labeled hippocampal stem cells did not differ between LRRK2 knock-out mice and wildtype littermate controls. Moreover, the number of surviving BrdU-positive hippocampal stem cells that can be detected 28 days after systemic BrdU administration showed no significant difference (data not shown). Importantly, by counting the numbers of neuroblasts expressing the early neuronal maturation marker doublecortin (DCX) we detected a significant (p<0.05) increase in the total number of DCX-immunopositive cells in the dentate gyrus of LRRK2 knockout mice compared to wildtype controls (4297±325 versus 2891±203 DCX-positive cells, mean ± SEM).

## Discussion

Prominent LRRK2 expression has been detected in the ventricular and subventricular zones as well as in the hippocampal formation of the embryonic and adult mouse brain. LRRK2 mRNA was also expressed in neurosphere cultures consisting of neuronal stem cells and precursor cells suggesting a role of LRRK2 in neurogenesis or in neurodevelopment [6], [7]. In the present study, wildtype and LRRK2+/− primary mouse ES cells were cultured followed by retinoic acid treatment to induce neuronal differentiation. This cell culture model has already been used to assess the effects of α-synuclein overexpression and amyloid precursor protein deficiency, respectively, on neuronal development [26], [27]. By immunocytochemical staining and microscopic evaluation no obvious differences in cell viability, proliferation, or neuronal differentiation was observed. Differential gene expression analysis however, revealed significant changes following retinoic acid-induced differentiation. The ES cell genome is transcriptionally hyperactive and undergoes large-scale silencing as cells differentiate [28] which was more prominent in the LRRK2+/− neural precursor cells. Notably, the central regulators of ES cell pluripotency, Lin28, Nanog, and Oct4, and several of their downstream pluripotency-linked target genes [21], [22] declined more rapidly in LRRK2+/− cells. On the other hand, expression of various voltage-gated ion channels and neurotransmitter receptors/transporters was increased in LRRK2-deficient neural precursor cells. Since both glutamatergic and GABAergic markers were upregulated to a similar extent, our data point to an acceleration of neuronal differentiation, and not to a change in neuronal phenotype in LRRK2+/− cells. Subtle phenotypic changes however, can only be excluded after extensive electrophysiological characterisation of the ES cell-derived neurons. Increased medium concentrations of GABA and glutamate in LRRK2+/− cell cultures may merely confirm accelerated neuronal differentiation. Non-synaptic GABA however, acts as a developmental signal on neural precursor cells promoting differentiation and maturation [29] which may also constitute a positive feedback loop in our cell culture system.

**Figure 8:**
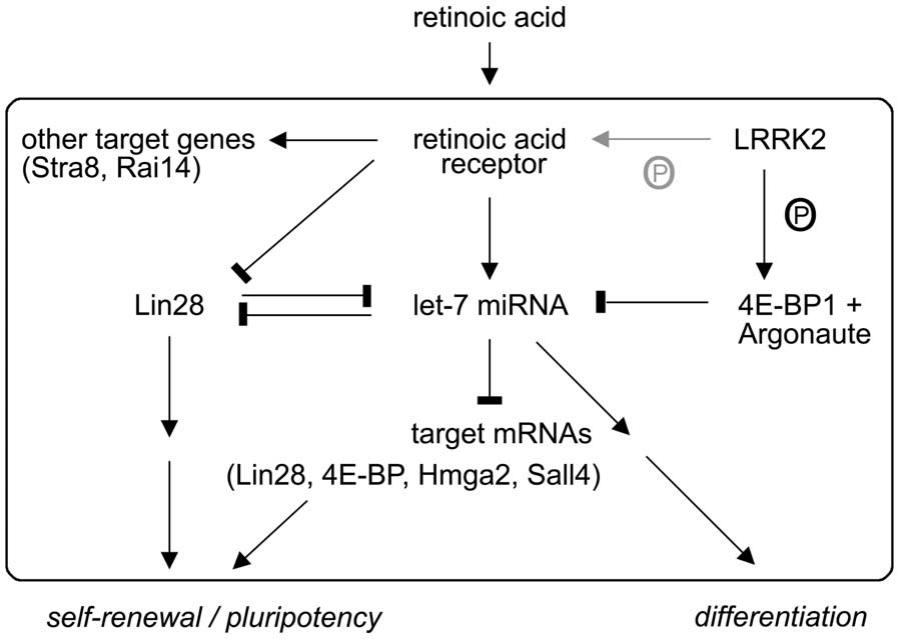
Model of how LRRK2-deficiency may promote retinoic-acid induced neuronal differentiation. Lin28 protein and let-7 miRNA are key regulators of pluripotency and differentiation, respectively. Lower protein levels of LRRK2 and 4E-BP1 in LRRK2+/− ES cells may alleviate suppression of let-7 miRNA function by LRRK2. LRRK2 may also phosphorylate retinoic acid receptors, thereby modulating transcriptional activation/repression of differentiation/stemness factors (for details see Discussion).

What may be the underlying mechanism for LRRK2 deficiency-mediated alterations in neuronal differentiation? LRRK2 phosphorylates moesin which regulates binding to F-actin, cytoskeletal stability, and neurite sprouting [23], [30], [31]. Moreover, actin cytoskeleton stability influences stem cell differentiation in culture [32], [33]. Moesin phosphorylation however, was unchanged in LRRK2-deficient differentiating neurons making a contribution unlikely. Since gene expression changes were most pronounced following retinoic acid treatment and since several genes containing retinoic acid response elements (e.g. Stra8, Rai14) were affected, we hypothesized that retinoic acid signaling may be altered in LRRK2+/− cells. In response to retinoic acid, mitogen-activated protein (MAP) kinases become activated which phosphorylate retinoic acid receptors, thereby modulating transcriptional activity and proteasomal degradation [25]. The kinase domain of LRRK2 exhibits sequence homology to the MAP kinase kinase kinase family, and there is *in vitro* evidence that LRRK2 acts within MAP kinase signaling cascades [34]. We could demonstrate that recombinant LRRK2 potently phosphorylates retinoic acid receptors *in vitro* indicating that LRRK2 may directly modulate its function (Figure 8). Cellular assays using retinoic acid-responsive element containing reporter genes are required to analyse the functional consequences *in vivo*.

Very recently, LRRK2 has been shown to antagonize let-7 miRNA-mediated translational repression in mature Drosophila neurons leading to neurodegeneration [35]. LRRK2 protein suppressed the inhibitory effects of let-7 miRNA by promoting the interaction of phosphorylated 4E-BP1 with Argonaute. In embryonic stem cells, let-7 miRNA acts as a key pro-differentiation factor by blocking mRNA translation of pluripotency factors, like Lin28, Sall4, and Hmga2 [36], [37]. In the self-renewing state, the RNA-binding protein Lin28 inhibits processing and maturation of let-7 miRNA. Administration of retinoic acid causes a repression of Lin28 mRNA and an upregulation of let-7 miRNA and a concomitant switch from self-renewal to differentiation [38]. In our study, repression of Lin28 and of several let-7 target mRNAs (4E-BP1, Sall4, Hmga2) was more pronounced in LRRK2-deficient ES cells after retinoic acid treatment. Thus, there is indirect evidence that lower protein levels of LRRK2 and 4E-BP1 in LRRK2+/− ES cells may alleviate suppression of let-7 miRNA function thereby accelerating retinoic acid-induced differentiation (Figure 8).

What may be the pathophysiological relevance of our findings in differentiating neurons to human Parkinson's disease patients carrying LRRK2 mutations? Although LRRK2 deficient/overexpressing mice do not exhibit a neurodegenerative phenotype, LRRK2 deficiency/overexpression in immature neurons in culture clearly affects neurite outgrowth [5], [39]. Interestingly, in a conditional transgenic mouse model of spinocerebellar ataxia type 1, expression of polyglutamine-expanded Ataxin-1 protein during five days of cerebellar Purkinje cell development is sufficient for the loss of motor coordination in adult mice [40]. Mutant Ataxin-1 destabilizes the transcription factor retinoic acid-related orphan receptor alpha leading to a reduced expression of retinoic acid-related orphan receptor alpha target genes that are critical for Purkinje cell development. Thus, a short-term disturbance of a retinoic acid receptor-coordinated neurodevelopmental program can result in neuronal dysfunction in the adult brain even in the absence of an immediate effect on neuronal phenotype. Besides its role in the developing nervous system, retinoic acid signaling is also required for maintenance of the nigrostriatal system [41]. The dopamine D2 receptor promotor contains a functional retinoic acid response element, and striatal D1/D2 receptor levels are reduced in retinoic acid receptor knock-out mice. Adult knock-out mice exhibit locomotor impairments in the absence of neuronal cell loss indicating that retinoic acid signaling defects may contribute to human PD [42]. Interestingly, knock-in mice expressing the PD-linked LRRK2(R1441C) mutation show impaired locomotor responses to the D2 receptor agonist quinpirole while the numbers and morphology of nigral dopaminergic neurons are unchanged [43]. Our gene expression profiling of basal ganglia tissue isolated from LRRK2 knock-out mouse brain did not reveal significant (adjusted p-value>0.05) changes in dopamine receptor mRNA expression compared to wildtype littermates. Altered expression of dopamine receptors however, may be restricted to subpopulations of neurons within single brain nuclei as has recently been shown in retinoid×receptor deficient mice [44]. Importantly, we detected an increased number of hippocampal neural progenitor cells in our LRRK2-deficient mice, while proliferation and survival of the hippocampal stem cell population was unchanged. Retinoic acid receptor signaling has been shown to influence neural precursor cell numbers in the mouse brain [41] indicating *in vivo* relevance of our findings in LRRK2+/− ES cells. Moreover, impairment of hippocampal neurogenesis has been described in animal models of memory loss and depression [45] suggesting that mutant LRRK2 may also contribute to non-motor symptoms of PD.

## Supporting Information

Figure S1 Southern Blot analysis of genomic DNA from transfected ES cells detects homologous recombination of the targeting vector at the mouse *LRRK2* locus in six heterozygous ES cell clones (15.3 kb) compared to wildtype cells (12.7 kb).
(TIF)

Figure S2 Ingenuity canonical pathway analysis of differentially-expressed mRNAs identified by microarray gene expression profiling. Significantly (p<0.005) enriched functional categories are depicted according to their p-value. The orange line indicates the calculated ratios. Blue bars represent the negative decadic logarithm of p-values based on Fisher's exact test.
(TIF)

Figure S3 Representative figure showing that membranes were routinely checked for protein load and protein transfer after blotting using the MemCode protein staining kit that reversibly stains for total protein.
(TIF)
