# Supplementary figures and images for: Leucine-Rich Repeat Kinase 2 Modulates Retinoic Acid-Induced Neuronal Differentiation of Murine Embryonic Stem Cells
Source: PLoS One. 2011 Jun 9;6(6):e20820. doi: 10.1371/journal.pone.0020820 (PMC3111438; doi:10.1371/journal.pone.0020820)

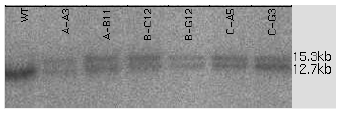

Supplement: Figure S1 — Southern Blot analysis of genomic DNA from transfected ES cells detects homologous recombination of the targeting vector at the mouse LRRK2 locus in six heterozygous ES cell clones (15.3 kb) compared to wildtype cells (12.7 kb). (TIF) [file pone.0020820.s001.tif]

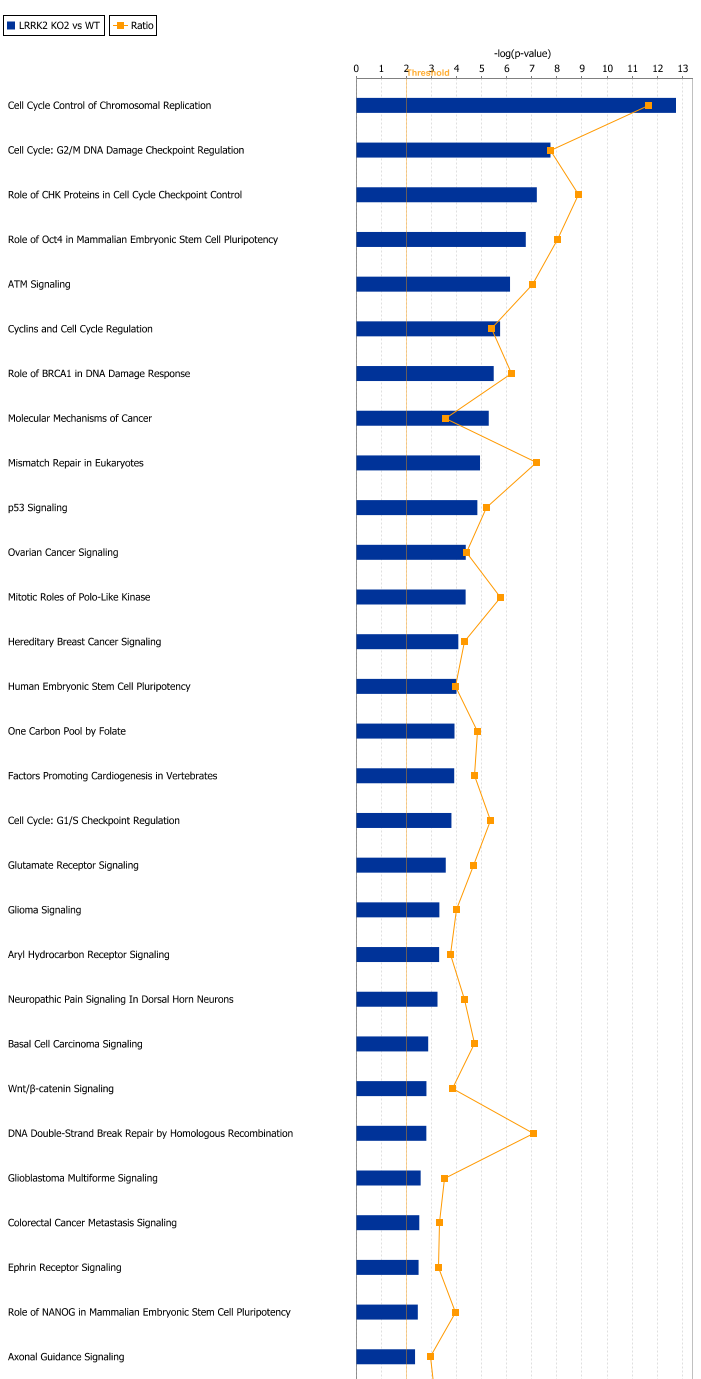

Supplement: Figure S2 — Ingenuity canonical pathway analysis of differentially-expressed mRNAs identified by microarray gene expression profiling. Significantly (p<0.005) enriched functional categories are depicted according to their p-value. The orange line indicates the calculated ratios. Blue bars represent the negative decadic logarithm of p-values based on Fisher's exact test. (TIF) [file pone.0020820.s002.tif]

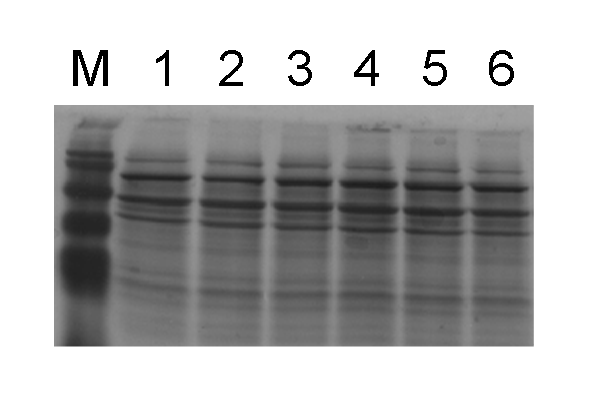

Supplement: Figure S3 — Representative figure showing that membranes were routinely checked for protein load and protein transfer after blotting using the MemCode protein staining kit that reversibly stains for total protein. (TIF) [file pone.0020820.s003.tif]
